# Association between dynamic change patterns of body mass or fat mass and incident stroke: results from the China Health and Retirement Longitudinal Study (CHARLS)

**DOI:** 10.3389/fcvm.2023.1269358

**Published:** 2023-11-21

**Authors:** Mengpi Lin, Shanting Zhou, Shanhong Gu

**Affiliations:** ^1^ Department of Neurology, Jieyang Ciyun Hospital, Jieyang, China; ^2^ Minzhi Community Health Centre, People’s Hospital of Shenzhen Longhua District, Shenzhen, China; ^3^ Department of Endocrinology, Shantou Hospital of Traditional Chinese Medicine, Shantou, China

**Keywords:** stroke, obesity, dynamic change, risk, primary prevention

## Abstract

**Objective:**

To assess the association between dynamic patterns of change in body mass or fat mass and stroke.

**Methods:**

A population-based cohort of participants was selected from the China Health and Retirement Longitudinal Study (CHARLS). Body mass and fat mass were measured using obesity-related indices, including weight, body mass index (BMI), waist circumference (WC), waist-to-height ratio (WHtR), lipid accumulation product (LAP), and visceral adiposity index (VAI). Five changed patterns were defined: low-stable, decreasing, moderate, increasing, and persistent-high. Logistic regression analysis was performed to evaluate the association between obesity-related indices and stroke.

**Results:**

A total of 5,834 participants were included, and the median age was 58.0 years. During a 7-years follow-up period, 354 (6.1%) participants developed stroke. The baseline levels of obesity-related indices were significantly associated with incident stroke. Regarding the dynamic change patterns, the low-stable pattern carried the lowest odds for stroke and the persistent-high pattern had the highest odds for stroke, with odds ratios of all the indices ranging from 1.73 to 3.37 (all *P *<* *0.05). The increasing pattern was also associated with a higher odds of stroke, whereas the moderate pattern of weight, BMI, and WHtR was comparable to the low-stable pattern in terms of stroke.

**Conclusion:**

Current status and dynamic changes in body mass and fat mass were significantly associated with incident stroke. Maintaining the low-stable pattern of body mass and fat mass as measured by weight, WC, BMI, WHtR, LAP, and VAI may be an alternative strategy for primary stroke prevention.

## 1. Introduction

Stroke is the second leading cause of premature death and disability and its incidence has been increased over the past three decades (1, 2). The burden of stroke has risen steadily since 1990, with 143 million loss of disability-adjusted life years, 6.55 million deaths due to stroke, and 101 million prevalent stroke cases in 2019 (3). Globally, 90.5% of the stroke burden is attributable to modifiable risk factors, including behavioral (such as poor diet and low physical activity) and metabolic (such as high body-mass index) factors (4). It suggests that stroke can be prevented to some extent by improving the lifestyle. Given the stroke's impact on quality of life and financial burden, the idea that prevention is always better than cure is desirable.

Among the risk factors for stroke, obesity has been the focus of attention, given the possibility of an obesity paradox. Several studies have reported an association between obesity and stroke; however, the results have been inconsistent. A Mendelian randomization study revealed that abdominal adiposity may lead to cerebrovascular diseases independent of blood pressure and glucose levels (5), whereas general obesity was not significantly associated with stroke (6). Previous studies were limited by methodological concerns, including the retrospective nature of most studies, the assessment of obesity using only body mass index (BMI), and potential confounding factors (7). In addition, most studies only used parameters from a single measurement, and few studies have assessed the effect of dynamic changes in obesity status on stroke. It has been shown that stable obesity and weight gain were associated with an increased mortality risk (8). Moreover, waist circumference (WC) increase is associated with higher mortality risk in middle-aged and older Chinese adults (9).

Given that the obesity status could change over time, the present study evaluated the association between dynamic change patterns of body mass or fat mass and the odds of stroke. Body mass or fat mass was measured using several commonly used obesity-related indices, including weight, WC, BMI, waist-to-height ratio (WHtR), lipid accumulation product (LAP), and visceral adiposity index (VAI).

## 2. Materials and methods

### 2.1. Participants selection

Study participants were selected from the China Health and Retirement Longitudinal Study (CHARLS), which is a nationally representative cohort. This project was supported by Peking University, and the details are available on its website (http://charls.pku.edu.cn/en). The CHARLS aims to collect a high-quality, nationally representative sample of Chinese residents aged 45 years and older to serve the needs of scientific research on middle-aged and older people. The baseline national wave of CHARLS was conducted in 2011, and 17,708 individuals from 28 provinces (150 counties/districts and 450 villages/residential committees) in China participated in the investigation. Three subsequent follow-ups were performed in 2013–2014 (wave 2), 2015–2016 (wave 3), and 2017–2018 (wave 4) and two blood samples were collected in waves 1 and 3.

**Figure 1:**
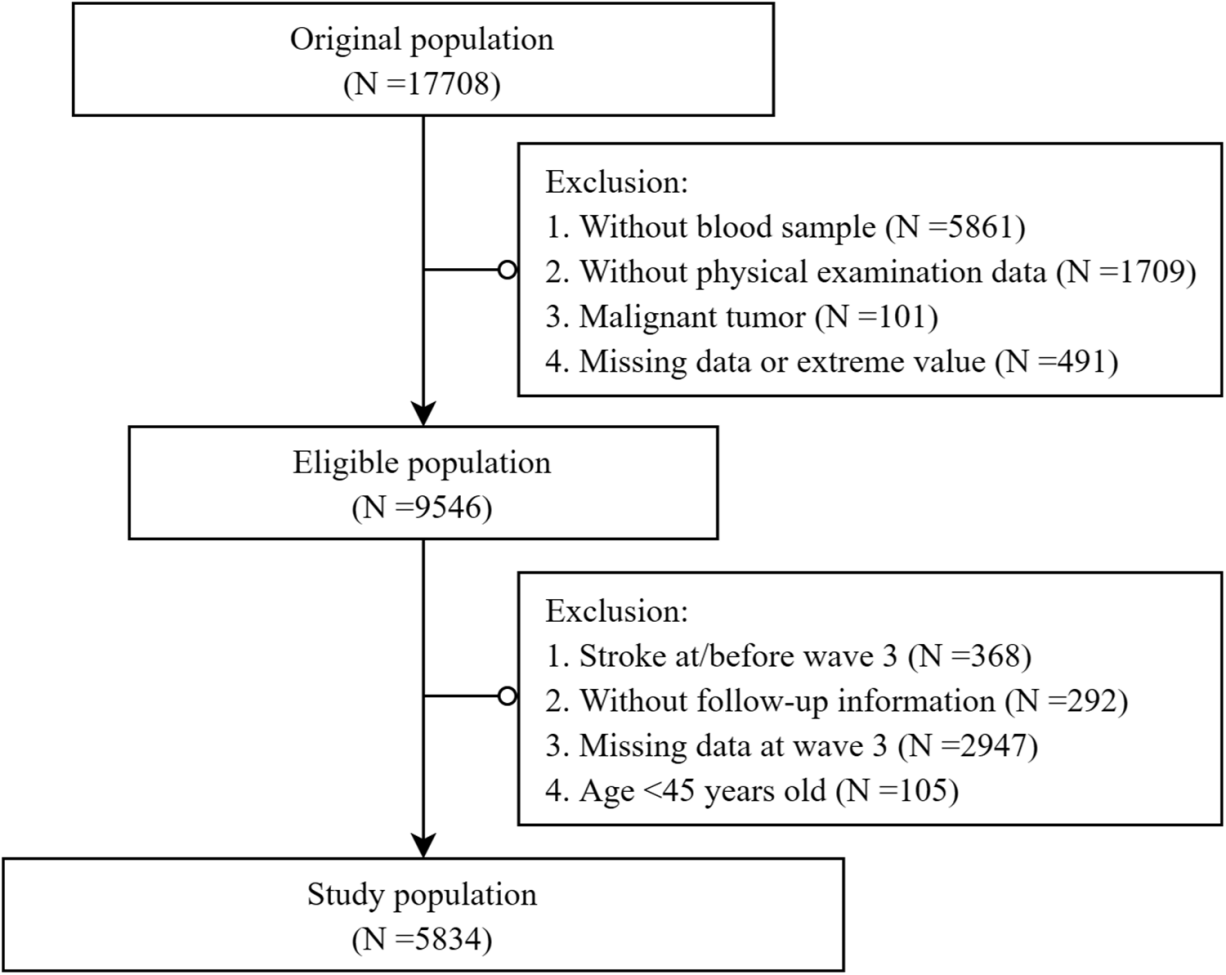
Flow chat of the study design.

The participants selection process is illustrated in Figure 1. Briefly, 9,546 individuals with complete data were screened for eligibility. For the present study, to evaluate the association between the dynamic change patterns of obesity status and the odds of stroke, those without a blood test at wave 3 were excluded. We also excluded participants who had experienced stroke events before/at wave 3 to minimize potential reverse causality. Finally, 5,834 participants were included (Figure 1). The CHARLS study received approval from the institutional review board of Peking University, and all participants provided written informed consent.

### 2.2. Data collection and definition

Information on demographics, socioeconomics, and health status was collected by trained investigators using computer-assisted personal interviews as described previously (10). The variables included in this study were age, sex, BMI, smoking and drinking consumption status, educational levels, systolic blood pressure (SBP), diastolic blood pressure (DBP), fasting plasma glucose (FPG), total cholesterol (TC), triglycerides (TG), low-density lipoprotein cholesterol (LDL-C), high-density lipoprotein cholesterol (HDL-C), serum creatinine (Scr), uric acid (UA), and high-sensitivity C reaction protein (hs-CRP). The chronic diseases included hypertension, diabetes mellitus (DM), and heart disease. Hypertension was defined as SBP ≥140 mmHg and/or DBP ≥90 mmHg or being under anti-hypertensive therapy. DM was defined as FPG ≥7.0 mmol/L or a history of DM diagnosed by a physician. Heart diseases included coronary heart disease, congestive heart failure and heart attacks.

### 2.3. Definition of exposure and outcome

The primary exposure was body mass or fat mass and its dynamic change patterns, measured using obesity-related indices including weight, WC, BMI, WHtR, LAP, and VAI. We first divided the study population into four groups according to the values of each index, with the first (Q1) and last (Q4) groups being those with values in lowest and highest quartiles, respectively. Five patterns were identified, based on the longitudinal trends of each index from the wave 1 to wave 3 surveys to evaluate dynamic changes in obesity status, including the low stable, decreasing, moderate, increasing, and persistent-high. Individuals with an index remaining in the lowest (Q1) or highest (Q4) quartiles in both surveys were identified as having low-stable or persistent-high patterns, respectively. Those remaining in Q2 and Q3 in both surveys were considered to have a moderate pattern. The decreasing group was defined as a decrease from a higher to a lower quartile, whereas the reverse was true for the definition of the increasing group. The outcome of the present study was an incident stroke during the wave 3 and wave 4 surveys, data on which was collected by asking a participant, “Have you been told by a doctor that you have been diagnosed with a stroke?” (11).

### 2.4. Statistical analyses

Data were presented as median (interquartile range) and number (percentage) where appropriate and were compared between the stroke and non-stroke groups using Mann–Whitney *U*-test or Chi-square test.

Given the same follow-up interval (from wave 3 to wave 4), logistic regression analysis was used to evaluate the association of obesity status and its dynamic change patterns with incident stroke. We first constructed an adjustment model (model 1) derived from directed acyclic graphs (DAGs) which generated minimal sufficient adjustment sets based on causal assumptions (Supplementary Figure S1). In addition, another two models were generated based on previous literature reports, clinical knowledge and collinearity testing. Model 2 was adjusted for demographic factors, physical and laboratory examinations, including age, sex, smoking and drinking consumption status, educational level, SBP, FPG, TC, LDL-C, Scr, UA, and hs-CRP levels. Model 3 further considered the disease status, including hypertension, DM, and heart disease. There was no significant collinearity among variables in the model 3. Odds ratios (OR) were reported for per SD increase of each index, quartile group (with Q1 serving as a reference), and change pattern (with the low-stable pattern serving as a reference). To response to reviewer's comments, we performed a sensitivity analysis by excluded those who developed hypertension or DM during the follow-up period. Statistical analyses were performed using SPSS 23.0 for Windows. A two-sided *P *<* *0.05 was considered statistically significant.

## 3. Results

### 3.1. Baseline characteristics

A total of 5,834 participants free of stroke at baseline were included in the final analysis, 54.5% of whom were women. The baseline characteristics of the study population are presented in Table 1. The median age was 58.0 years, and BP and FPG were 127/74 mmHg and 5.7 mmol/L, respectively. The percentages of patients with hypertension and DM were 39.5% and 15.9%, respectively. During a 7-years follow-up period, 354 (6.1%) participants developed stroke. Compared to the non-stroke group, those who developed a stroke were older and had higher BP, FPG, TG, and hs-CRP levels. The proportions of patients with hypertension, DM and heart disease were higher in the stroke group than in the non-stroke group. All six obesity-related indices, including weight, WC, BMI, WHtR, LAP, and VAI, were higher in the stroke group than in the non-stroke group (Table 1).

**Table 1:** Baseline characteristics of the study population.

Characteristics	Overall (*n *=* *5,834)	Non-stroke (*n *=* *5,480)	Stroke (*n *=* *354)	*P* value
Age (year)	58.0 (52.0–64.0)	58.0 (51.0–64.0)	60.0 (55.0–66.0)	<0.001
Sex, women, *n* (%)	3,179 (54.5)	2,974 (54.3)	205 (57.9)	0.183
Smoking, *n* (%)	2,226 (38.2)	2,088 (38.1)	138 (39.0)	0.741
Drinking, *n* (%)	2,363 (40.5)	2,214 (40.4)	149 (42.1)	0.530
Education, *n* (%)				
Primary and below	4,105 (70.4)	3,838 (70.0)	267 (75.4)	
Middle school	1,194 (20.5)	1,135 (20.7)	59 (16.7)	0.096
High school and above	535 (9.2)	507 (9.3)	28 (7.9)	
Systolic blood pressure (mmHg)	126.7 (114.3–141.7)	126.3 (114.0–140.7)	134.0 (119.7–149.7)	<0.001
Diastolic blood pressure (mmHg)	74.3 (67.3–83.0)	74.3 (67.0–83.0)	78.3 (70.3–87.0)	<0.001
Fasting plasma glucose (mmol/L)	5.7 (5.3–6.3)	5.7 (5.2–6.2)	5.8 (5.3–6.5)	0.001
Total cholesterol (mmol/L)	5.0 (4.3–5.6)	4.9 (4.3–5.6)	5.1 (4.4–5.7)	0.031
Triglycerides (mmol/L)	1.2 (0.8–1.7)	1.2 (0.8–1.7)	1.3 (0.9–1.8)	<0.001
LDL-C (mmol/L)	3.0 (2.4–3.6)	3.0 (2.4–3.6)	3.0 (2.4–3.7)	0.135
HDL-C (mmol/L)	1.3 (1.1–1.6)	1.3 (1.1–1.6)	1.2 (1.0–1.5)	0.001
Serum creatinine (µmol/L)	65.9 (56.9–76.9)	65.9 (56.9–76.9)	66.9 (57.9–76.9)	0.329
Uric acid (mg/dl)	4.2 (3.5–5.1)	4.2 (3.5–5.1)	4.3 (3.5–5.2)	0.455
hs-CRP (mg/L)	1.00 (0.54–2.06)	0.97 (0.54–2.02)	1.24 (0.67–2.54)	<0.001
Hypertension, *n* (%)	2,305 (39.5)	2,094 (38.2)	211 (59.6)	<0.001
Diabetes, *n* (%)	925 (15.9)	843 (15.4)	82 (23.2)	<0.001
Heart diseases, *n* (%)	669 (11.5)	600 (10.9)	69 (19.5)	<0.001
Adiposity indices				
Weight (kg)	58.0 (51.0–66.0)	58.0 (51.0–66.0)	60.0 (53.0–68.0)	0.001
Waist circumference (cm)	85.0 (78.0–92.0)	85.0 (78.0–92.2)	89.0 (82.0–96.0)	<0.001
Body mass index (kg/m^2^)	23.2 (21.0–25.9)	23.2 (21.0–25.8)	24.0 (21.7–26.8)	<0.001
Waist-to-height ratio	0.55 (0.50–0.59)	0.55 (0.50–0.59)	0.57 (0.53–0.61)	<0.001
Lipid accumulation product	27.5 (15.2–47.9)	26.9 (14.9–47.2)	34.3 (21.2–56.6)	<0.001
Visceral adiposity index	1.50 (0.89–2.60)	1.48 (0.88–2.58)	1.80 (1.12–2.87)	<0.001

Data are present as median (interquartile range) or *n* (%). LDL-C: low-density lipoprotein cholesterol; HDL-C: high-density lipoprotein cholesterol; hs-CRP: high-sensitive C-reactive protein.

### 3.2. Association between baseline obesity-related indices and stroke

**Figure 2:**
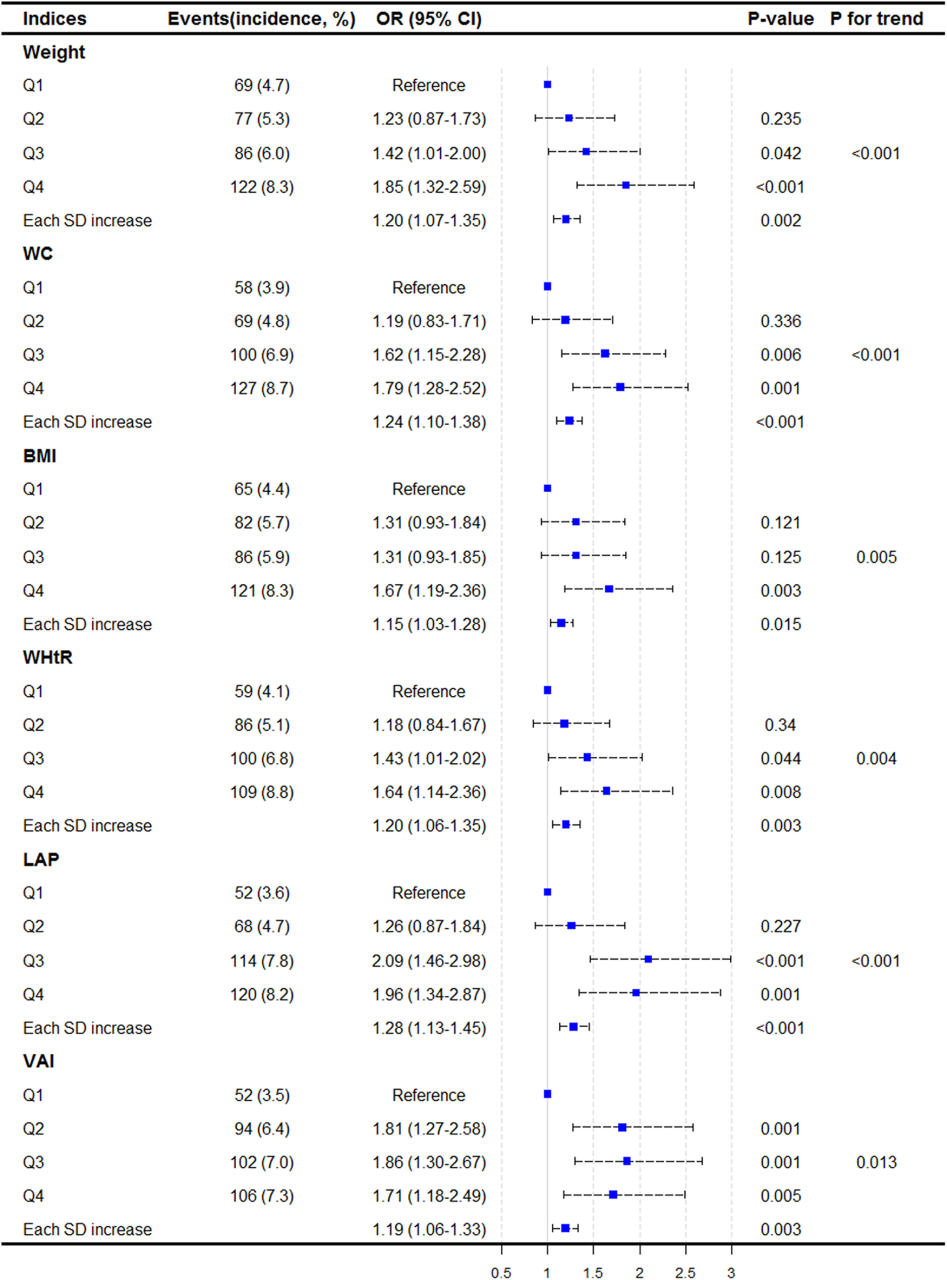
Association between baseline adiposity indices and incident stroke. Results are shown as odds ratios (95% CI) derived from logistic regression models adjusted for age, sex, smoking, drinking, educational levels, SBP, FPG, TC, LDL-C, Scr, UA, hs-CRP, history of hypertension, diabetes, and heart diseases.

The cutoff points and numbers of participants for each quartile group of obesity-related indices are shown in Supplementary Table S1. The incidence of stroke increased with each index, and the fourth quartile had the highest incidence of stroke. After adjusting for age, sex, smoking and alcohol consumption status, educational level, SBP, FPG, TC, LDL, Scr, UA, hs-CRP, and history of hypertension, DM, and heart diseases, all obesity indices were positively associated with the odds of stroke (Figure 2). Compared with the lowest quartiles, the highest quartiles had 64%–96% increased odds of incident stroke (all *P* < 0.05). Each SD increase was associated with a 15%–28% increased odds of stroke (all *P *< 0.05).

### 3.3. Association of change patterns of body mass or fat mass with stroke

Details of the sample distribution of the five change patterns of the indices are listed in Supplementary Table S1. The persistent-high pattern of each index was associated with the highest stroke incident rate. Univariate analysis revealed that compared with the low-stable pattern, the other four patterns had a significantly higher odds of incident stroke (Table 2). Similar results were obtained when adjusted for variables selected by DAGs analysis (Table 2, Model 1). The results did not change substantially after adjusting for demographic factors and physical and laboratory examination results (Table 2, Model 2). When disease status was further considered (Table 2, Model 3), the association between obesity-related indices and stroke was attenuated but remained significant. The odds ratios for the persistent-high patterns of all indices ranged from 1.73 to 3.37 (all *P* < 0.05). The increasing pattern had a higher odds of stroke, whereas the moderate pattern of weight, BMI, and WHtR showed odds of stroke comparable to the low-stable pattern. Sensitivity analysis by excluded those who developed hypertension or DM during the follow-up period yielded consistent results (Supplementary Table S2).

**Table 2:** Association between adiposity index changes and incident stroke.

Change patterns	Crude model		Model 1		Model 2		Model 3	*P* value
	OR (95% CI)	*P* value	OR (95% CI)	*P* value	OR (95% CI)	*P* value	OR (95% CI)	
Weight								
Low stable	Reference		Reference		Reference		Reference	
Decreasing	1.61 (1.09–2.40)	0.018	1.47 (0.97–2.23)	0.067	1.71 (1.13–2.57)	0.010	1.58 (1.05–2.38)	0.028
Moderate	1.21 (0.86–1.69)	0.282	1.25 (0.87–1.78)	0.227	1.40 (0.99–2.00)	0.059	1.33 (0.94–1.90)	0.111
Increasing	1.35 (0.91–2.00)	0.140	1.44 (0.96–2.17)	0.077	1.60 (1.07–2.40)	0.023	1.52 (1.02–2.28)	0.042
Persistent high	1.93 (1.38–2.72)	<0.001	1.70 (1.15–2.50)	0.007	2.26 (1.57–3.27)	<0.001	1.93 (1.33–2.81)	0.001
WC								
Low stable	Reference		Reference		Reference		Reference	
Decreasing	1.81 (1.17–2.80)	0.008	1.47 (0.94–2.30)	0.090	1.63 (1.05–2.53)	0.031	1.55 (1.00–2.42)	0.052
Moderate	1.81 (1.19–2.77)	0.006	1.59 (1.03–2.45)	0.036	1.76 (1.14–2.70)	0.010	1.70 (1.11–2.61)	0.016
Increasing	1.53 (1.01–2.32)	0.047	1.44 (0.94–2.21)	0.093	1.55 (1.01–2.36)	0.043	1.50 (0.98–2.29)	0.061
Persistent high	2.80 (1.87–4.21)	<0.001	1.88 (1.21–2.92)	0.005	2.45 (1.61–3.73)	<0.001	2.14 (1.40–3.27)	<0.001
BMI								
Low stable	Reference		Reference		Reference		Reference	
Decreasing	2.11 (1.40–3.18)	<0.001	1.74 (1.13–2.68)	0.012	2.03 (1.33–3.09)	0.001	1.88 (1.23–2.88)	0.004
Moderate	1.30 (0.89–1.91)	0.172	1.25 (0.84–1.86)	0.263	1.41 (0.95–2.08)	0.087	1.35 (0.91–1.99)	0.137
Increasing	1.80 (1.22–2.65)	0.003	1.79 (1.20–2.67)	0.004	1.95 (1.31–2.90)	0.001	1.88 (1.27–2.80)	0.002
Persistent high	2.26 (1.56–3.27)	<0.001	1.74 (1.15–2.64)	0.009	2.32 (1.56–3.44)	<0.001	1.99 (1.34–2.98)	0.001
WHtR								
Low stable	Reference		Reference		Reference		Reference	
Decreasing	1.53 (0.96–2.44)	0.074	1.15 (0.71–1.86)	0.579	1.31 (0.81–2.12)	0.266	1.23 (0.76–1.98)	0.405
Moderate	1.62 (1.07–2.47)	0.024	1.29 (0.83–1.99)	0.259	1.51 (0.99–2.33)	0.059	1.41 (0.91–2.17)	0.121
Increasing	1.70 (1.13–2.56)	0.011	1.45 (0.95–2.22)	0.086	1.63 (1.07–2.47)	0.023	1.55 (1.02–2.35)	0.042
Persistent high	2.56 (1.68–3.90)	<0.001	1.51 (0.94–2.42)	0.086	2.00 (1.27–3.15)	0.003	1.73 (1.10–2.74)	0.018
LAP								
Low stable	Reference		Reference		Reference		Reference	
Decreasing	2.55 (1.51–4.31)	<0.001	2.09 (1.20–3.66)	0.010	2.51 (1.47–4.30)	0.001	2.32 (1.35–3.98)	0.002
Moderate	3.23 (1.94–5.38)	<0.001	2.78 (1.63–4.74)	<0.001	3.18 (1.88–5.38)	<0.001	2.99 (1.77–5.07)	<0.001
Increasing	2.63 (1.60–4.31)	<0.001	2.40 (1.44–4.02)	0.001	2.73 (1.64–4.53)	<0.001	2.57 (1.55–4.27)	<0.001
Persistent high	4.19 (2.54–6.92)	<0.001	2.71 (1.49–4.92)	0.001	3.92 (2.29–6.72)	<0.001	3.37 (1.96–5.80)	<0.001
VAI								
Low stable	Reference		Reference		Reference		Reference	
Decreasing	1.65 (1.04–2.63)	0.035	1.03 (0.59–1.78)	0.930	1.54 (0.95–2.49)	0.078	1.44 (0.89–2.33)	0.139
Moderate	2.20 (1.40–3.47)	0.001	1.55 (0.93–2.58)	0.095	2.10 (1.31–3.37)	0.002	1.99 (1.24–3.20)	0.004
Increasing	1.69 (1.08–2.63)	0.021	1.34 (0.83–2.18)	0.235	1.69 (1.06–2.68)	0.027	1.60 (1.01–2.55)	0.046
Persistent high	2.31 (1.45–3.69)	<0.001	1.02 (0.53–1.95)	0.963	2.02 (1.22–3.35)	0.006	1.78 (1.07–2.96)	0.027

*Note.* Results are shown as odds ratios (95% CI) derived from logistic regression models. Model 1 was adjusted for variables selected by DAG analysis, including age, sex, smoking, SBP, DBP, FPG, TC, TG, LDL-C, HDL-C, Scr, UA, hs-CRP, hypertension, diabetes, and heart diseases. Model 2 adjusted for age, sex, smoking, drinking, education, SBP, FPG, TC, LDL-C, Scr, UA, and hsCRP. Model 3 was adjusted for variable in model 2 plus history of hypertension, diabetes, and heart diseases.

## 4. Discussion

The present study, based on a nationally representative cohort with a median follow-up period of 7 years, demonstrates that current status and dynamic changes in body mass and fat mass are significantly associated with incident stroke. For immediate status, those in the highest quartile had a 64%–96% increased odds of stroke compared with those in the lowest quartile of each obesity-related index, including weight, WC, BMI, WHtR, LAP, and VAI. In addition, a persistent-high pattern for each of the six indices indicated the highest odds of stroke development. Moreover, an increasing pattern also increases the stroke odds.

Although the association between obesity and cardiovascular disease has been widely reported, the impact of dynamic changes in body mass or fat mass on health status has only recently come into focus. In the Chinese population, stable obesity across adulthood, weight gain from young to middle adulthood and weight loss from middle to late adulthood were associated with an increased mortality risk (8). Another study conducted in the United States showed that weight loss from obesity to overweight between early adulthood and mid-life appeared to be associated with a reduction in mortality risk compared to persistent obesity, suggesting a benefit of weight loss for obese individuals (12). More recently, Hussain et al. revealed that weight loss and WC decrease were associated with an increase in all-cause mortality (13). Several studies have reported an association between weight change and stroke with inconsistent results. A study from Japan showed that weight gain during middle age was associated with an increased risk of stroke in women (14). In contrast, results from Norway revealed that weight gain during early life but not mid-life was associated with an increased risk of stroke in healthy men (15). In the present study, conducted in Chinese middle-aged and older populations, decreasing, increasing, and persistent-high patterns of weight change were associated with a significantly higher odds of stroke, suggesting that maintaining low- or moderate-stable weight patterns after middle age may be most beneficial for reducing stroke risk. Similar results were found in a recent study in which weight loss did not reduce the risk of stroke in obese individuals (16).

In the present study, changes in BMI, WC and WHtR had similar effects on stroke and body weight. Several studies have reported an association between changes in WC and mortality; however, the conclusions remained inconsistent (17–20). Yuan et al. recently reported that both gain and loss in WC were associated with a higher mortality risk compared to the stable group (9), similar to our study that focused on the outcome of stroke. Therefore, maintaining a low- to moderate-stable pattern of WC levels, rather than increasing or decreasing them, may be beneficial for preventing stroke. To the best of our knowledge, the effects of visceral fat changes on stroke have rarely been evaluated. Our study measured fat accumulation using LAP and VAI: the immediate level of visceral fat and dynamic patterns were significantly associated with stroke. As expected, the low-stable pattern was associated with the lowest odds of stroke. In addition, among the other dynamic patterns, including the decreasing, moderate, increasing, and persistent-high patterns, the decreasing pattern had a lower odds of stroke development, indicating a potential benefit for stroke prevention by reducing visceral fat in those with moderate to high levels. The impact of visceral adiposity has been demonstrated to be greater than that of general and abdominal obesity (21, 22). Future studies should focus on the changes of body weight and WC and further explore the relationship between the change pattern of visceral fat and stroke and other cardiovascular diseases.

The strengths of the present study include the use of a nationally representative cohort with a prospective design that allowed us to evaluate the association between dynamic changes in body mass and fat mass and the odds of stroke and the fact that a series of confounding factors were considered to provide insights for the primary prevention of stroke. This study has some limitations that warrant further discussion. First, the changed patterns of obesity-related indices were based on quartile groups owing to the lack of a standard cut-off value, and we could only provide qualitative rather than quantitative results. In addition, data-driven methods, such as growth mixture modeling or latent class models, would be better for defining temporal patterns. Secondly, the interval between the two measurements was relatively short, and the effect of long-term changes on the outcome could not be assessed. However, a short-term change pattern may be more appropriate for risk assessment and intervention in a large sample population. Third, we failed to distinguish between intentional and unintentional changes in obesity status. Future studies should focus on the reasons for these changes in developing appropriate intervention strategies. It is also important to consider both diet and exercise parameters simultaneously. Finally, although adjusted for a series of known confounders, other unmeasured factors should be further considered in future studies.

In conclusion, not only immediate status but also dynamic changes in body mass and fat mass are significantly associated with incident stroke. Maintaining the low-stable pattern of body mass and fat mass as measured by weight, BMI, WHtR, LAP, and VIA may be an alternative strategy for primary stroke prevention. For those with moderate levels, maintaining stability and decreasing variation (rather than increasing or decreasing) may have the best benefits, and an appropriate reduction is recommended for those with high obesity-related indices.

## Data Availability

The original contributions presented in the study are included in the article/**Supplementary Material**, further inquiries can be directed to the corresponding author. Data of the China Health and Retirement Longitudinal Study are available at its website (http://charls.pku.edu.cn/).
