# Supplementary material for: Association between dynamic change patterns of body mass or fat mass and incident stroke: results from the China Health and Retirement Longitudinal Study (CHARLS)
Source: Front Cardiovasc Med. 2023 Nov 21;10:1269358. doi: 10.3389/fcvm.2023.1269358 (PMC10702727; doi:10.3389/fcvm.2023.1269358)
Supplement: Supplementary file 1 [file Table1.docx]

**Association between** **dynamic change patterns of body mass or fat mass and risk of stroke: A nationally representative cohort study**

**Running title:** Obesity and stroke

**All authors’ name:** Mengpi Lin^1^, Shanting Zhou^2^, Shanhong Gu^3^

**Authors’ affiliations:**

^1^ Department of Neurology, Jieyang Ciyun Hospital, Jieyang, Guangdong, China

^2^ People’s Hospital of Shenzhen Longhua District, Shenzhen, Guangdong, China

^3^ Department of Endocrinology, Shantou Hospital of Traditional Chinese Medicine, Shantou, Guangdong, China

**Correspondence to:** Shanhong Gu, Shantou Hospital of Traditional Chinese Medicine, No. 3 Shaoshan Road, Shantou, Guangdong, 515000, China. Email: [314995431@qq.com](mailto:314995431@qq.com)

**Table S1 Sample distribution and incident rate of stroke**

| **Indices** | **Quartile (range)** | | | | |  | **Change pattern** | | | | |
| --- | --- | --- | --- | --- | --- | --- | --- | --- | --- | --- | --- |
|  |  | **Number**  **(n)** | **Events**  **(n)** | **Incidence (%)** | ***P* value** |  | **Patterns** | **Number**  **(n)** | **Events**  **(n)** | **Incidence (%)** | ***P* value** |
| Weight | Q1 (men: ≤54; women: ≤49.5) | 1488 | 69 | 4.6 | < 0.001 |  | Low stable | 1209 | 54 | 4.5 | 0.001 |
|  | Q2 (men: 54.1-60.8; women: 49.6-55.9) | 1481 | 77 | 5.2 |  |  | Decreasing | 713 | 50 | 7.0 |  |
|  | Q3 (men: 60.9-68.4; women: 56.0-63.2) | 1480 | 88 | 5.9 |  |  | Moderate | 1837 | 98 | 5.3 |  |
|  | Q4 (men: ≥68.5; women: ≥63.3) | 1490 | 123 | 8.3 |  |  | Increasing | 844 | 50 | 5.9 |  |
|  |  |  |  |  |  |  | Persistent high | 1231 | 102 | 8.3 |  |
| WC | Q1 (men: ≤78.0; women: ≤78.9) | 1514 | 60 | 4.0 | < 0.001 |  | Low stable | 919 | 32 | 3.5 | < 0.001 |
|  | Q2 (men: 78.1-84.2; women: 79.0-85.5) | 1477 | 69 | 4.7 |  |  | Decreasing | 1028 | 63 | 6.1 |  |
|  | Q3 (men: 84.3-91.3; women: 85.6-92.4) | 1473 | 101 | 6.9 |  |  | Moderate | 1222 | 75 | 6.1 |  |
|  | Q4 (men: ≥91.4; women: ≥92.5) | 1475 | 127 | 8.6 |  |  | Increasing | 1532 | 80 | 5.2 |  |
|  |  |  |  |  |  |  | Persistent high | 1133 | 104 | 9.2 |  |
| BMI | Q1 (≤21.04) | 1485 | 65 | 4.4 | < 0.001 |  | Low stable | 1104 | 42 | 3.8 | < 0.001 |
|  | Q2 (21.05-23.22) | 1485 | 84 | 5.7 |  |  | Decreasing | 740 | 57 | 7.7 |  |
|  | Q3 (23.23-25.86) | 1487 | 86 | 5.8 |  |  | Moderate | 1652 | 81 | 4.9 |  |
|  | Q4 (≥25.87) | 1482 | 122 | 8.2 |  |  | Increasing | 1129 | 75 | 6.6 |  |
|  |  |  |  |  |  |  | Persistent high | 1209 | 99 | 8.2 |  |
| WHtR | Q1 (≤0.49) | 1474 | 61 | 4.1 | < 0.001 |  | Low stable | 843 | 31 | 3.7 | < 0.001 |
|  | Q2 (0.50-0.54) | 1725 | 86 | 5.0 |  |  | Decreasing | 834 | 46 | 5.5 |  |
|  | Q3 (0.55-0.59) | 1495 | 101 | 6.8 |  |  | Moderate | 1456 | 85 | 5.8 |  |
|  | Q4 (≥0.60) | 1245 | 109 | 8.8 |  |  | Increasing | 1723 | 105 | 6.1 |  |
|  |  |  |  |  |  |  | Persistent high | 978 | 87 | 8.9 |  |
| LAP | Q1 (≤15.24) | 1484 | 52 | 3.5 | < 0.001 |  | Low stable | 831 | 19 | 2.3 | < 0.001 |
|  | Q2 (15.25-27.48) | 1485 | 69 | 4.6 |  |  | Decreasing | 1031 | 58 | 5.6 |  |
|  | Q3 (27.49-47.68) | 1485 | 115 | 7.7 |  |  | Moderate | 1083 | 76 | 7.0 |  |
|  | Q4 (≥47.69) | 1485 | 121 | 8.1 |  |  | Increasing | 1814 | 105 | 5.8 |  |
|  |  |  |  |  |  |  | Persistent high | 1075 | 96 | 8.9 |  |
| VAI | Q1 (≤0.89) | 1484 | 52 | 3.5 | < 0.001 |  | Low stable | 717 | 25 | 3.5 | 0.002 |
|  | Q2 (0.90-1.50) | 1490 | 96 | 6.4 |  |  | Decreasing | 1244 | 70 | 5.6 |  |
|  | Q3 (1.51-2.60) | 1486 | 102 | 6.9 |  |  | Moderate | 1167 | 86 | 7.4 |  |
|  | Q4 (≥2.61) | 1479 | 107 | 7.2 |  |  | Increasing | 1810 | 104 | 5.7 |  |
|  |  |  |  |  |  |  | Persistent high | 896 | 69 | 7.7 |  |

**Table S2 Association between adiposity index changes and incident stroke^#^**

| **Change patterns** | **Crude model** | |  | **Model 1** | |  | **Model 2** | |  | **Model 3** | |
| --- | --- | --- | --- | --- | --- | --- | --- | --- | --- | --- | --- |
|  | **OR (95% CI)** | ***P* value** |  | **OR (95% CI)** | ***P* value** |  | **OR (95% CI)** | ***P* value** |  | **OR (95% CI)** | ***P* value** |
| **Weight** |  |  |  |  |  |  |  |  |  |  |  |
| Low stable | Reference |  |  | Reference |  |  | Reference |  |  | Reference |  |
| Decreasing | 1.86 (1.21-2.86) | 0.005 |  | 1.81 (1.16-2.83) | 0.009 |  | 1.96 (1.26-3.05) | 0.003 |  | 1.78 (1.14-2.78) | 0.011 |
| Moderate | 1.33 (0.91-1.93) | 0.140 |  | 1.44 (0.97-2.14) | 0.067 |  | 1.54 (1.05-2.27) | 0.029 |  | 1.44 (0.98-2.13) | 0.064 |
| Increasing | 1.44 (0.93-2.22) | 0.103 |  | 1.60 (1.02-2.50) | 0.042 |  | 1.69 (1.08-2.64) | 0.021 |  | 1.58 (1.01-2.47) | 0.044 |
| Persistent high | 2.18 (1.50-3.17) | < 0.001 |  | 2.17 (1.43-3.29) | < 0.001 |  | 2.50 (1.67-3.74) | < 0.001 |  | 2.05 (1.36-3.09) | 0.001 |
| **WC** |  |  |  |  |  |  |  |  |  |  |  |
| Low stable | Reference |  |  | Reference |  |  | Reference |  |  | Reference |  |
| Decreasing | 1.94 (1.21-3.10) | 0.006 |  | 1.63 (1.01-2.64) | 0.047 |  | 1.74 (1.08-2.79) | 0.024 |  | 1.63 (1.01-2.63) | 0.046 |
| Moderate | 1.73 (1.09-2.75) | 0.021 |  | 1.55 (0.96-2.48) | 0.072 |  | 1.67 (1.04-2.67) | 0.033 |  | 1.59 (0.99-2.55) | 0.054 |
| Increasing | 1.69 (1.07-2.65) | 0.023 |  | 1.56 (0.99-2.47) | 0.058 |  | 1.69 (1.07-2.67) | 0.024 |  | 1.62 (1.02-2.55) | 0.040 |
| Persistent high | 3.16 (2.04-4.89) | < 0.001 |  | 2.32 (1.45-3.71) | < 0.001 |  | 2.71 (1.72-4.26) | < 0.001 |  | 2.27 (1.44-3.59) | < 0.001 |
| **BMI** |  |  |  |  |  |  |  |  |  |  |  |
| Low stable | Reference |  |  | Reference |  |  | Reference |  |  | Reference |  |
| Decreasing | 1.99 (1.28-3.09) | 0.002 |  | 1.74 (1.09-2.75) | 0.019 |  | 1.88 (1.19-2.97) | 0.007 |  | 1.72 (1.09-2.72) | 0.021 |
| Moderate | 1.26 (0.94-1.89) | 0.269 |  | 1.25 (0.82-1.90) | 0.310 |  | 1.34 (0.88-2.04) | 0.171 |  | 1.26 (0.83-1.91) | 0.288 |
| Increasing | 1.79 (1.18-2.71) | 0.006 |  | 1.78 (1.16-2.74) | 0.008 |  | 1.92 (1.26-2.93) | 0.003 |  | 1.81 (1.18-2.77) | 0.006 |
| Persistent high | 2.41 (1.63-3.57) | < 0.001 |  | 2.04 (1.32-3.16) | 0.001 |  | 2.39 (1.57-3.63) | < 0.001 |  | 1.96 (1.28-2.99) | 0.002 |
| **WHtR** |  |  |  |  |  |  |  |  |  |  |  |
| Low stable | Reference |  |  | Reference |  |  | Reference |  |  | Reference |  |
| Decreasing | 1.55 (0.92-2.60) | 0.098 |  | 1.23 (0.72-2.09) | 0.455 |  | 1.32 (0.78-2.25) | 0.302 |  | 1.22 (0.71-2.07) | 0.471 |
| Moderate | 1.85 (1.17-2.92) | 0.009 |  | 1.55 (0.97-2.50) | 0.070 |  | 1.72 (1.08-2.76) | 0.023 |  | 1.56 (0.98-2.51) | 0.064 |
| Increasing | 1.84 (1.17-2.90) | 0.008 |  | 1.59 (1.00-2.55) | 0.050 |  | 1.76 (1.11-2.79) | 0.017 |  | 1.64 (1.03-2.61) | 0.036 |
| Persistent high | 2.92 (1.84-4.64) | < 0.001 |  | 1.92 (1.15-3.20) | 0.012 |  | 2.26 (1.38-3.72) | 0.001 |  | 1.88 (1.14-3.09) | 0.014 |
| **LAP** |  |  |  |  |  |  |  |  |  |  |  |
| Low stable | Reference |  |  | Reference |  |  | Reference |  |  | Reference |  |
| Decreasing | 2.72 (1.51-4.90) | 0.001 |  | 2.32 (1.25-4.31) | 0.008 |  | 2.67 (1.47-4.85) | 0.001 |  | 2.40 (1.31-4.37) | 0.004 |
| Moderate | 3.77 (2.14-6.64) | < 0.001 |  | 3.36 (1.86-6.06) | < 0.001 |  | 3.71 (2.07-6.62) | < 0.001 |  | 3.39 (1.89-6.08) | < 0.001 |
| Increasing | 2.99 (1.72-5.19) | < 0.001 |  | 2.87 (1.62-5.09) | < 0.001 |  | 3.11 (1.77-5.47) | < 0.001 |  | 2.85 (1.62-5.03) | < 0.001 |
| Persistent high | 4.76 (2.73-8.33) | < 0.001 |  | 3.36 (1.74-6.47) | < 0.001 |  | 4.32 (2.38-7.85) | < 0.001 |  | 3.55 (1.94-6.48) | < 0.001 |
| **VAI** |  |  |  |  |  |  |  |  |  |  |  |
| Low stable | Reference |  |  | Reference |  |  | Reference |  |  | Reference |  |
| Decreasing | 1.93 (1.16-3.21) | 0.012 |  | 1.27 (0.70-2.30) | 0.434 |  | 1.80 (1.07-3.05) | 0.028 |  | 1.65 (0.98-2.80) | 0.061 |
| Moderate | 2.42 (1.46-4.01) | 0.001 |  | 1.79 (1.02-3.13) | 0.044 |  | 2.32 (1.38-3.91) | 0.002 |  | 2.15 (1.27-3.63) | 0.004 |
| Increasing | 1.80 (1.10-2.95) | 0.020 |  | 1.51 (0.88-2.57) | 0.132 |  | 1.81 (1.09-3.02) | 0.023 |  | 1.69 (1.01-2.82) | 0.046 |
| Persistent high | 2.52 (1.50-4.23) | < 0.001 |  | 1.25 (0.62-2.53) | 0.536 |  | 2.16 (1.24-3.77) | 0.007 |  | 1.85 (1.05-3.24) | 0.032 |

^#^ Sensitivity analysis by excluded those who developed hypertension or diabetes during the follow-up period (n =535). Results are shown as odds ratios (95% CI) derived from logistic regression models. Model 1 was adjusted for variables selected by DAG analysis, including age, sex, smoking, SBP, DBP, FPG, TC, TG, LDL-C, HDL-C, Scr, UA, hs-CRP, hypertension, diabetes, and heart diseases. Model 2 adjusted for age, sex, smoking, drinking, education, SBP, FPG, TC, LDL-C, Scr, UA, and hsCRP. Model 3 was adjusted for variable in model 2 plus history of hypertension, diabetes, and heart diseases.


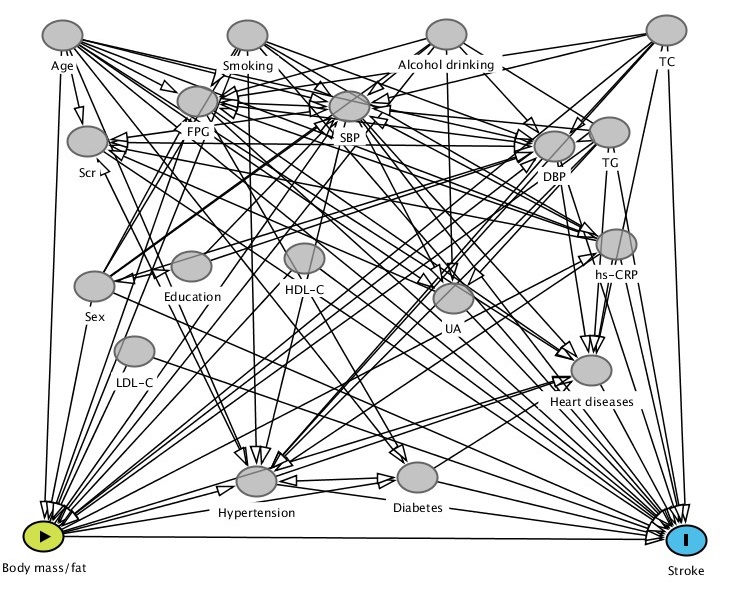


**Figure S1** Directed acyclic graph for selecting confounding factors
